# Growth differentiation factor 15 level changes in the early and late periods after radiofrequency and pulsed field catheter ablation of atrial fibrillation

**DOI:** 10.3389/fcvm.2026.1798578

**Published:** 2026-08-05

**Authors:** Barbora Steklá, Květa Pelinková, Hana Benáková, Jan Šimek, Milan Dusík, Aleš Linhart, Štěpán Havránek

**Affiliations:** 1 2nd Department of Medicine—Department of Cardiovascular Medicine of the 1st Faculty of Medicine, Charles University and General University Hospital in Prague, Prague, Czechia; 2 Institute of Medical Biochemistry and Laboratory Diagnostics of the 1st Faculty of Medicine, Charles University and General University Hospital in Prague, Prague, Czechia

**Keywords:** atrial fibrillation, bleeding score, catheter ablation, GDF-15, pulsed-field ablation

## Abstract

**Introduction:**

GDF-15 (growth differentiation factor 15) is a protein from the transforming growth factor β (TGF-*β*) cytokine family. In patients with atrial fibrillation (AF), it is a potent marker of bleeding adverse events in anticoagulated patients and a predictor of overall mortality. However, its level is affected by various conditions, including radiofrequency catheter ablation (RFCA). The study aimed to describe the dynamics of GDF-15 following a novel AF treatment approach, pulsed field ablation (PFA).

**Methods:**

This prospective observational study enrolled 50 patients [median (IQR) age 63 (53; 71) years, 62% males] undergoing PFA and compared them with 50 patients [65 (57; 69) years, 64% males] post-RFCA.

**Results:**

In the PFA group, 30 (60%) patients had paroxysmal AF, compared with 20 (40%) patients in the RFCA group. The median (IQR) GDF-15 level at baseline was 943 (733; 1,148) ng/L. The median value recorded 24 h after PFA was 1,363 (901; 1,867) ng/L. No significant difference in values was observed between patients treated with RFCA and those treated with PFA. However, when comparing GDF-15 values in paroxysmal AF separately, significantly higher post-procedural GDF-15 levels (immediately post-ablation and in 24 h after procedure) were observed after PFA than RFCA: 963 (672; 1,266) vs. 630 (506; 804) ng/L with *p* < 0.001 and 1,363 (901; 1,960) vs. 843 (710; 1,195) ng/L, *p* < 0.01, respectively. No correlation was found between GDF-15 levels and procedural data.

**Conclusion:**

GDF-15 levels are significantly affected by catheter ablation of AF, regardless of the ablation technology used.

## 1 Introduction

Growth differentiation factor 15 (GDF-15) is a member of the transforming growth factor *β* (TGF-*β*) cytokine superfamily. Under normal physiological conditions, it is present at low levels in various tissues, including the liver, kidneys, heart, and lungs. Its biological role is not yet fully defined. GDF-15 is involved in metabolic regulation, modulation of inflammatory pathways, cellular repair and growth processes, and control of apoptosis (1).

GDF-15 has emerged as a strong biomarker associated with bleeding complications in patients receiving anticoagulant therapy (2, 3). It has also been identified as a predictor of all-cause mortality in individuals with atrial fibrillation (AF) (3). Several risk scores have been developed to help assess the probability of bleeding, with the HAS-BLED (4) and the ORBIT (5) scores being the most widely used. However, their predictive accuracy is limited, which reduces their clinical utility. To address these limitations, a newer model, the ABC-bleeding risk score (where ABC stands for Age, Biomarkers, and Clinical history), was introduced. Among the most significant predictors of bleeding events included in the score were GDF-15, hemoglobin, high-sensitivity cardiac troponin T (hs-cTnT), age, and a history of bleeding (2). This model has shown improved predictive performance compared to HAS-BLED and ORBIT scores (2, 6).

Catheter ablation is a well -established therapeutic option for selected patients with AF (7). Myocardial injury caused by thermal ablation, including radiofrequency catheter ablation (RFCA), leads to increased concentrations of cardiac injury biomarkers (8, 9) and affects markers involved in inflammation and coagulation pathways (8). Pulsed-field ablation (PFA) is a novel approach in the treatment of AF. PFA is based on high-energy, ultrashort electrical impulses that cause nonthermal cell death (10). PFA is, at least, non-inferior in the head-to-head clinical comparison with thermal ablation (9).

It is already known that RFCA results in significant changes in GDF-15 levels (11). However, data on the behavior of GDF-15 after PFA are currently lacking. To our knowledge, no studies have yet described the dynamics of GDF-15 in response to PFA or performed a direct comparison across different ablation technologies. Additionally, it remains unclear how post-PFA changes in GDF-15 correlate with the kinetics of other routinely used biomarkers.

Despite the fact that the PFA is a safe and highly effective tool, it is often associated with intravascular hemolysis (12) or elevation of various biomarkers (10). The primary objective was to characterize changes in GDF-15 levels following PFA of AF. The secondary objective was to compare these changes with RFCA. Secondary objectives also included evaluation of changes in other biomarkers, including hs-cTnT and N-terminal pro-B-type natriuretic peptide (NT-proBNP), and assessment of short- and long-term changes in the ABC-bleeding score.

## 2 Methods

This was a single-center prospective observational study of patients undergoing PFA of AF with a comparison to a historical cohort of patients who underwent RFCA of AF in a previously published study conducted at the same institution (11).

These patients underwent six sequential blood sample collections to analyze GDF-15 levels and other commonly used biomarkers in predefined time windows. Patients were enrolled at the General University Hospital in Prague between April 2023 and May 2024. The study was conducted in accordance with Good Clinical Practice and in compliance with the principles outlined in the Declaration of Helsinki. The local Ethics Committee approved the study protocol. Individual written consent was obtained from each patient.

### 2.1 Patient selection

We enrolled patients with paroxysmal and persistent AF who were indicated for catheter ablation. The exclusion criteria were: 1) refusal to participate in the study, 2) conditions that contraindicate catheter ablation, 3) impossibility of long-term follow-up, 4) conditions that lead to an increase in GDF-15 level, except for cardiac ones: malignant diseases, pregnancy, renal insufficiency with creatinine clearance under 30 mL/min.

### 2.2 Catheter ablation

The pulsed-field (PF) catheter ablation was performed under general anesthesia. In patients on direct oral anticoagulants, only the morning dose on the day of the procedure was omitted. The international normalized ratio (INR) was targeted between 2 and 3 in patients on vitamin K antagonists. Femoral venous access was achieved using ultrasound control. According to local standards, the procedure was guided by intracardiac echocardiography (ACUSON AcuNav catheter, Biosense-Webster). A decapolar coronary sinus catheter was inserted only in patients with persistent AF or when cavotricuspid isthmus ablation was planned. Transseptal puncture was initially performed using a transseptal sheath SL 1 (Abbott). This sheath was thereafter replaced with a 13-F deflectable transseptal sheath (Faradrive, Boston Scientific). Intravenous heparin was administered to maintain an activated clotting time greater than 300 s throughout the procedure. The ablation was performed using a pentaspline catheter (Farawave 31 mm, Boston Scientific) and a PFA generator (Farastar, Boston Scientific). Pulmonary vein isolation was performed in all patients. An ablation using a biphasic bipolar waveform was performed as follows: four applications with the ablation catheter in the “basket” configuration and four applications with the ablation catheter in the “flower” configuration for each pulmonary vein ostium. When an entrance or exit block was not achieved, extra PF energy applications were administered. At the operator's discretion, additional ablation lesions were performed. Further lesion sets involved the application of overlapping PF pairs using a pentaspline catheter, targeting regions such as the posterior left atrial wall between the contralateral pulmonary veins, the mitral isthmus, or the cavotricuspid isthmus.

### 2.3 Follow-up and medical treatment after catheter ablation

During regular follow-up visits at 3-month intervals, symptoms and relevant clinical events were documented, and a standard electrocardiogram (ECG) was recorded. Standard ECG monitoring was performed in all patients between the third and sixth months post-ablation. Anticoagulant treatment was administered based on current practice. After ablation, all the patients received anticoagulation therapy for at least 3 months. The subsequent treatment extension was at the treating physician's discretion, based on the CHA_2_DS_2_-VASc/CHA_2_DS_2_-VA score used at the time of the study. All class Ic or III antiarrhythmic drugs were discontinued at Month 3. Persistent arrhythmia, if observed at Month 3, was electrically cardioverted. In the event of arrhythmia recurrence, antiarrhythmic drugs were initiated, and a repeat catheter ablation was considered. The follow-up protocol was intentionally designed to mirror the methodology of the previous study, thereby enabling direct comparison of biomarker dynamics between the two cohorts (11).

### 2.4 Study procedures

Clinical examinations, echocardiography, ECG, and routine laboratory tests were performed regularly on all enrolled patients. Enrolled PFA patients underwent six subsequent blood samples in predefined time intervals, as depicted in Table 1. The first two samplings were performed via peri-procedurally inserted sheaths, with the first sheath inserted at the beginning and the second removed at the end of the procedure. The third sampling was conducted on the first post-ablation day before hospital discharge. The last samples were collected during outpatient check-ups at 90 days, 6 months, and 12 months. The timing of each sample corresponded to the previous report on the effect of RFCA (11), with two exceptions: RFCA patients underwent one laboratory test 48 h post-ablation, and the last test was performed after 6 months.

**Table 1:** Laboratory testing in patients with pulsed field catheter ablation.

Blood sample	Biomarkers
**# 1:** Beginning of ablation – baseline	GDF-15, NT-proBNP, hs-cTnT
**# 2:** End of ablation—0 h after ablation	GDF-15, hs-cTnT
**# 3:** First post-ablation day (20–24 h after ablation)	GDF-15, hs-cTnT
**# 4:** 90 days after ablation	GDF-15, NT-proBNP
**# 5:** 6 months after ablation	GDF-15, NT-proBNP
**# 6:** 12 months after ablation	GDF-15, NT-proBNP

GDF-15, growth differentiation factor 15; hs-cTnT, high-sensitivity cardiac Troponin T; NT-proBNP, N-terminal pro-B-type natriuretic peptide.

Blood samples were taken in K_2_-EDTA tubes and stored frozen until analyzed in batches within 6 months of collection. Analyses were performed on the Roche COBAS e411 analyzer at the Institute of Medical Biochemistry and Laboratory Diagnostics, First Faculty of Medicine, Charles University in Prague, and the General University Hospital in Prague. GDF-15 concentrations were determined using the Roche Diagnostics GDF-15 assay. NT-proBNP and hs-cTnT concentrations were measured using commercially available Roche assays on the same analyzer. To minimize measurement bias, all laboratory analyses were performed in the same laboratory using standardized assays. Biomarker sampling was performed according to a predefined protocol. When comparing PFA and RFCA groups, the analyses were based on identical time points whenever available. To estimate bleeding risk in patients undergoing catheter ablation of AF, we determined the ABC-bleeding score using biomarker levels measured peri-procedurally. According to Hijazi et al. (2), the ABC-bleeding score incorporates age, GDF-15, hs-cTnT, hemoglobin, and prior bleeding. The methodology was identical to the previous study (11).

### 2.5 Statistical analysis

Continuous variables were expressed as median with interquartile (25th–75th percentile) range (IQR); or ranges (minimum-maximum) are provided where appropriate. Categorical variables were expressed as n (%). All study objectives were analyzed using standard statistical methods, including the Mann–Whitney U test, the Fisher test, and Spearman's correlation, as appropriate. Absolute GDF-15 levels were set as the primary endpoint. Changes in GDF-15 levels, assessment of longitudinal GDF-15 dynamics across available time points, and calculation of the area under the curve (AUC) during the first 24 h after ablation were defined as secondary endpoints. The results in patients with PFA were compared with those of a historical cohort of patients with RFCA (11). Repeated measures ANOVA was applied to adjust between-subjects and within-subjects effects (sphericity test and Greenhouse-Geisser test) in appropriate situations. A multivariate regression analysis of determinants of GDF-15 increase in 24 h was used. As this was an exploratory study, no formal sample size calculation was performed. The sample size was determined by the number of consecutive eligible patients undergoing PFA during the study period and by the availability of a comparable RFCA cohort. A *p*-value <0.05 was considered statistically significant. Statistical analyses were performed with Statistica version 12 software (StatSoft, Inc., Tulsa, USA), MedCalc Statistical Software, Version 19.7 (MedCalc Software, Ostend, Belgium; 2021), RStudio 2022.07.2 + 576 [RStudio Team (2020). RStudio: Integrated Development for R. RStudio, PBC, Boston, MA, URL: http://www.rstudio.com/].

## 3 Results

We enrolled 50 patients with a median (IQR) age of 63 (53; 71) years, 62% male, who were planned for PFA of AF. A total of 53 patients were initially enrolled, of whom 3 withdrew consent, leaving 50 patients included in the final analysis; no other condition led to termination. Baseline clinical and demographical data are in Table 2, PFA group.

**Table 2:** Baseline clinical and demographic data.

Parameter	PFA group *n* = 50	RFCA group *n* = 50	P PFA vs. RFCA
Age (years)	63 (53; 71)	65 (57; 69)	0.14
Male sex	31 (62%)	32 (64%)	0.84
Type of AF			
- Paroxysmal	30 (60%)	20 (40%)	0.07
- Non-paroxysmal	20 (40%)	30 (60%)	0.07
Heart failure	14 (28%)	16 (32%)	0.67
- Reduced ejection fraction	9 (18%)	11 (22%)	0.62
- Preserved ejection fraction	5 (10%)	5 (10%)	1.00
Arterial hypertension	30 (60%)	36 (72%)	0.21
Dyslipidaemia	31 (62%)	30 (60%)	0.84
Diabetes mellitus type 2	4 (8%)	9 (18%)	0.14
Coronary artery disease	2 (4%)	5 (10%)	0.25
Vascular disease	1 (2%)	1 (2%)	1.00
Stroke/Transitory ischemic attack	4 (8%)	3 (6%)	0.70
Major bleeding	0 (0%)	0 (0%)	-
Minor bleeding	1 (2%)	2 (4%)	0.57
Body mass index (kg/m^2^)	29 (25; 33)	31 (28; 36)	0.06
Indexed left atrial volume (mL/m^2^)	37 (32; 44)	43 (35; 50)	**0**.**04**
Left ventricular ejection fraction (%)	63 (57; 66)	62 (56; 65)	0.59
Left ventricular enddiastolic diameter (mm)	50 (47; 52)	51 (47; 55)	0.19
CHA_2_DS_2_-VASc score	2 (1; 3)	2 (1; 4)	0.08
HAS-BLED score	0.5 (0; 1)	2 (1; 2)	**< 0.001**
ABC-bleeding score	0.98 (0.66; 1.34)	1.37 (0.84; 1.84)	**0**.**006**
Antiarrhythmic drugs			
- Beta blockers	39 (78%)	36 (72%)	0.49
- Propafenone	20 (40%)	12 (24%)	0.09
- Amiodarone	11 (22%)	25 (50%)	**0**.**004**
- Sotalol	1 (2%)	1 (2%)	1.00
- Verapamil	1 (2%)	0 (0%)	0.33
Antithrombotic drugs before ablation			
- Warfarin	1 (2%)	2 (4%)	0.57
- Apixaban	18 (36%)	14 (28%)	0.40
- Rivaroxaban	22 (44%)	22 (44%)	1.00
- Dabigatran	8 (16%)	11 (22%)	0.45
- Other	0 (0%)	1 (2%)	0.33
History of cardioversion	27 (54%)	29 (58%)	0.69

Values are expressed as median (IQR) or n (%). NS, Not significant; AF, Atrial fibrillation; PFA, pulsed field ablation; RFCA, radiofrequency catheter ablation.

Bold values indicate statistically significant results (*p* < 0.05).

### 3.1 PFA procedural data

Pulmonary vein isolation was completed in all patients in the PFA group. Six PFA patients (12%) also underwent cavotricuspid isthmus ablation. Additional ablation lesions in the left atrium (LA), except for pulmonary vein isolation and cavotricuspid isthmus, were made in 17 (34%) patients in the PFA group. More detailed information is in Table 3, PFA group.

**Table 3:** Procedural data.

Parameter	PFA group *n* = 50	RFCA group *n* = 50	P PFA vs. RFCA
Pulmonary vein isolation	50 (100%)	48 (96%)	0.16
Ablation cavotricuspid isthmus	6 (12%)	3 (6%)	0.30
Additional ablation in the left atrium	17 (34%)	24 (48%)	0.16
Procedure time (min)	73 (60; 90)	155 (130; 180)	**< 0.001**
Radiofrequency time (min)	-	40 (33; 49)	-
Number of PFA applications	54 (40; 66)	-	-
Complications	0 (0%)	3 (6%)	0.08

Values are expressed as median (IQR) or *n* (%). NS, not significant; PFA, pulsed field ablation; RFCA, radiofrequency catheter ablation.

Bold values indicate statistically significant results (*p* < 0.05).

### 3.2 GDF-15 values after the PFA

**Figure 1:**
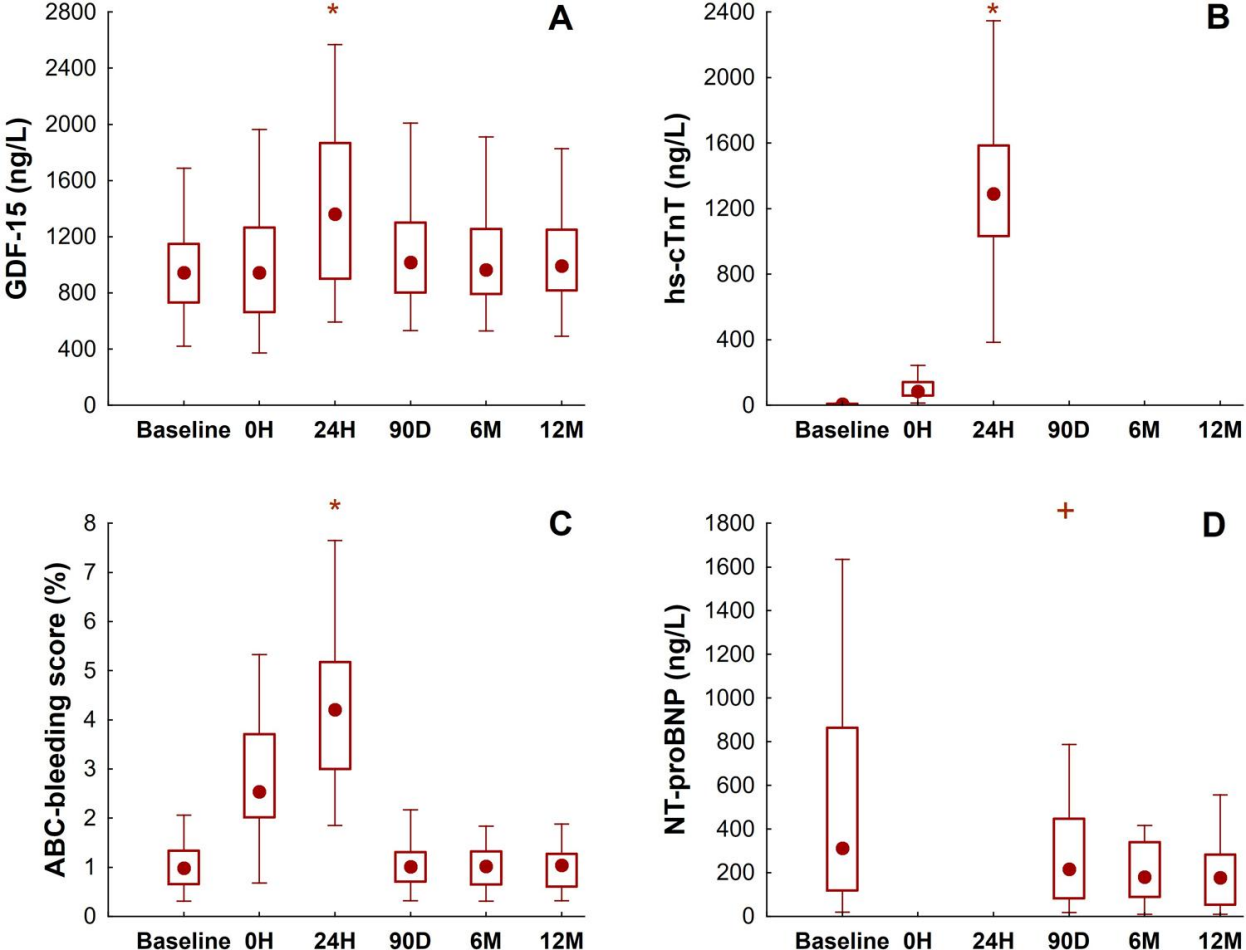
Dynamics of biomarkers and ABC bleeding score post PFA. Panel **(A)** Dynamics of Growth Differentiation Factor 15 (GDF-15). Panel **(B)** Dynamics of high-sensitivity cardiac Troponin T (hs-cTnT). Panel **(C)** Dynamics of ABC bleeding score. Panel **(D)** Dynamics of N-terminal B-type natriuretic propeptide (NT-proBNP). H, hours; D, days; M, months. The box-and-whisker plot shows the median, IQR, and range, excluding outliers. Asterisks indicate *p* < 0.001, and crosses indicate *p* < 0.05 (compared to the baseline value).

**Figure 2:**
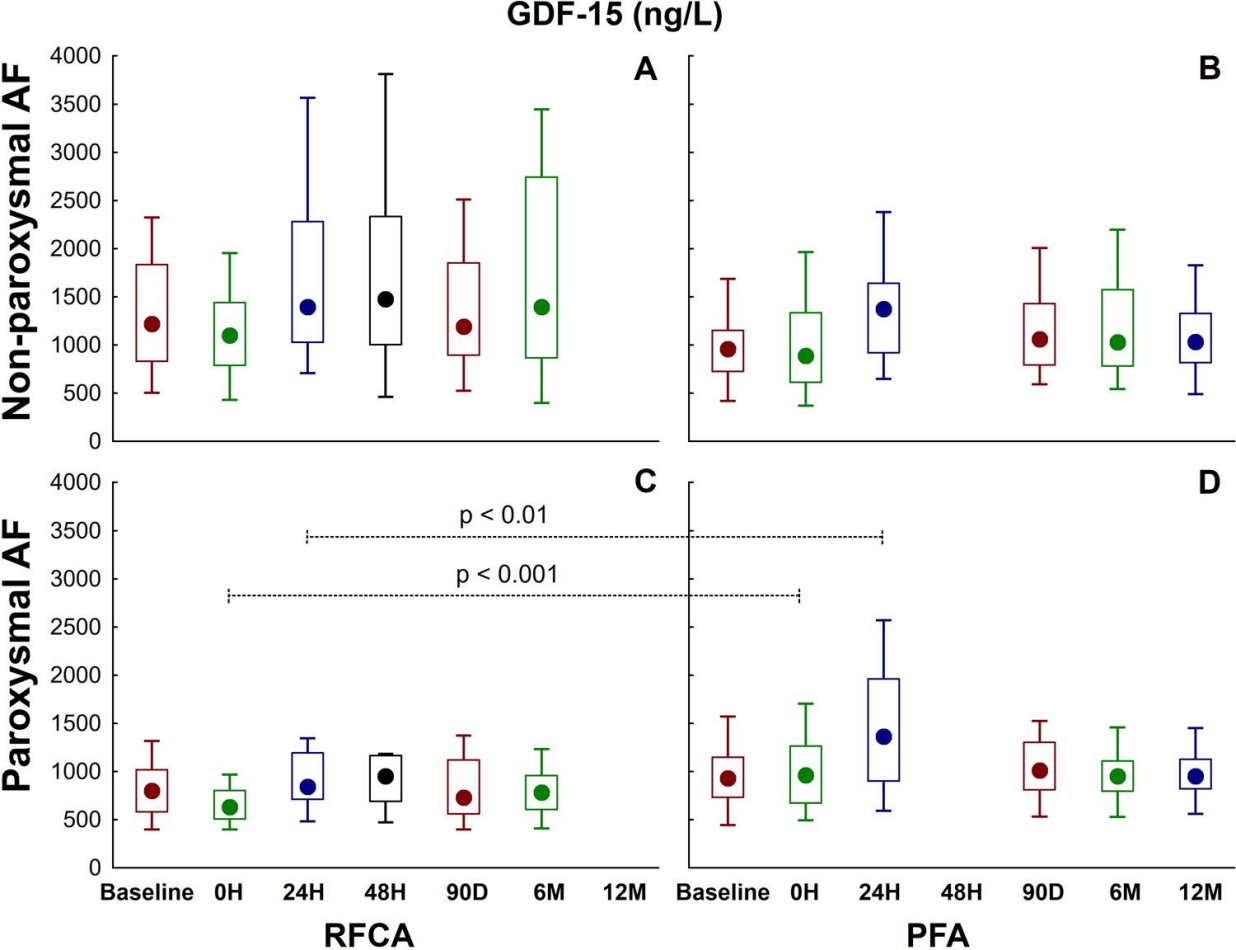
Dynamics of GDF-15, comparison of RFCA and PFA. AF, atrial fibrillation; GDF-15, growth differentiation factor 15; NS, not significant; RFCA, radiofrequency catheter ablation; PFA, pulsed field ablation. The box-whiskers plot shows median, IQR, and range without outliers.

The baseline GDF-15 level was 943 (733; 1,148) ng/L. The dynamics of GDF-15 levels are visualized in Figure 1A. The median value of GDF-15 after 24 h post-ablation reached 1,363 (901; 1,867) ng/L, which was significantly higher than baseline (*p* < 0.001). Values of GDF-15 at 90 days, 6 months, and 12 months did not differ from the baseline level. No statistical significance between corresponding GDF-15 levels was found when comparing patients with paroxysmal and non-paroxysmal AF, Figures 2B,D.

### 3.3 Correspondence of GDF-15 levels to hs-cTnT and NT-proBNP levels after the PFA

**Figure 3:**
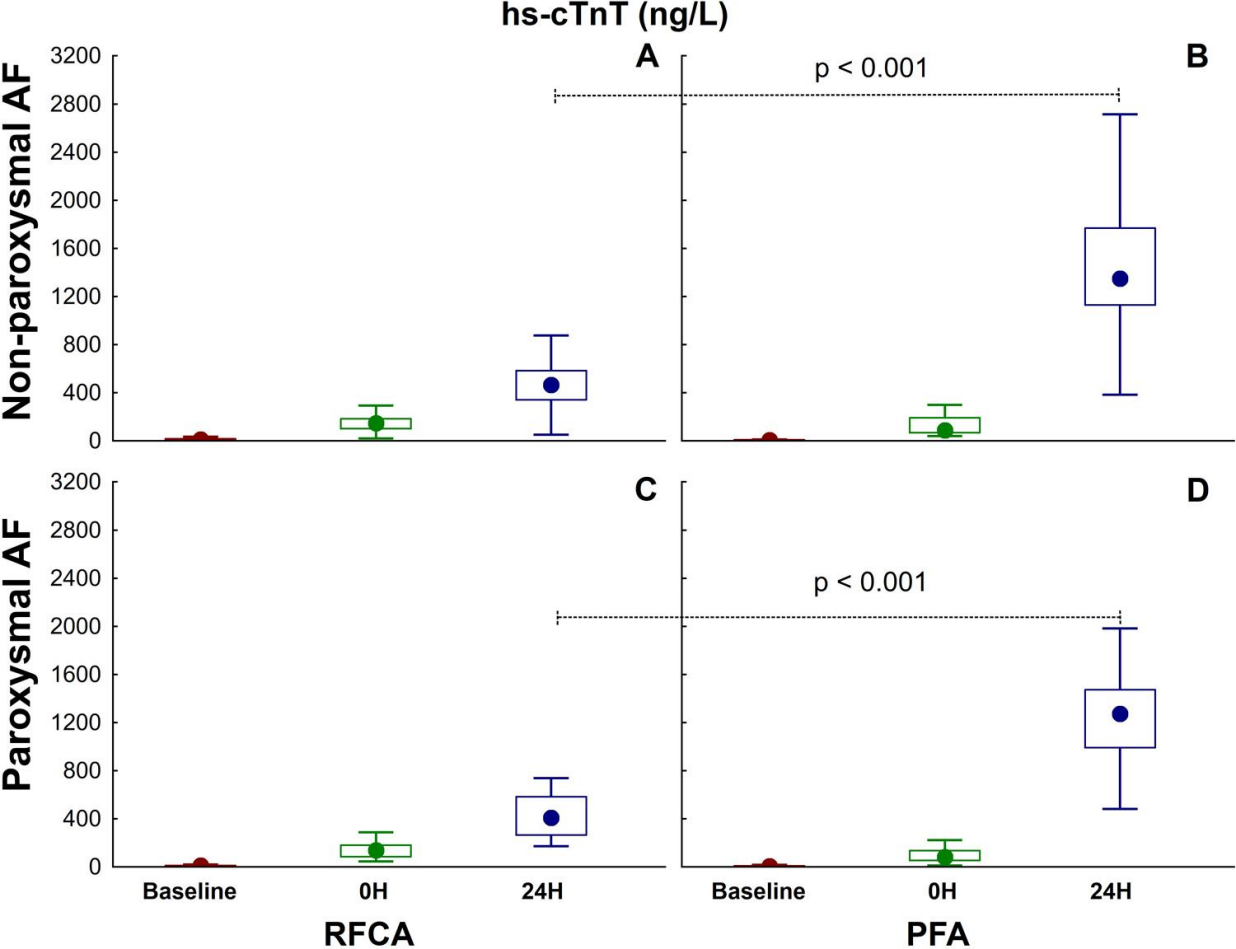
Dynamics of hs-cTnT, comparison of RFCA and PFA. AF, atrial fibrillation; hs-cTnT – high-sensitivity cardiac troponin T; NS, not significant; RFCA, radiofrequency catheter ablation; PFA, pulsed field ablation. The box-whiskers plot shows median, IQR, and range without outliers.

The baseline hs-cTnT plasma level was 8 (6; 10) ng/L in PFA. The median value of hs-cTnT after 24 h post-ablation increased up to 1,292 (1,032; 1,585) ng/L, which was significantly higher than baseline (*p* < 0.001). The dynamics of hs-cTnT levels post PFA are shown in Figure 1B. No significant difference was found between patients with paroxysmal and non-paroxysmal AF at the corresponding hs-cTnT levels (Figures 3B,D). Only a weak correlation between GDF-15 and hs-cTnT was found in patients with paroxysmal AF (R = 0.31; *p* = 0.045). In the remaining patients, no correlation was identified.

In correlation with changes in GDF-15 and hs-cTnT levels post-PFA, we observed changes in the ABC bleeding score (Figure 1C). The score increased significantly 24 h after catheter ablation. At 3 months, observed values were comparable to the baseline.

**Figure 4:**
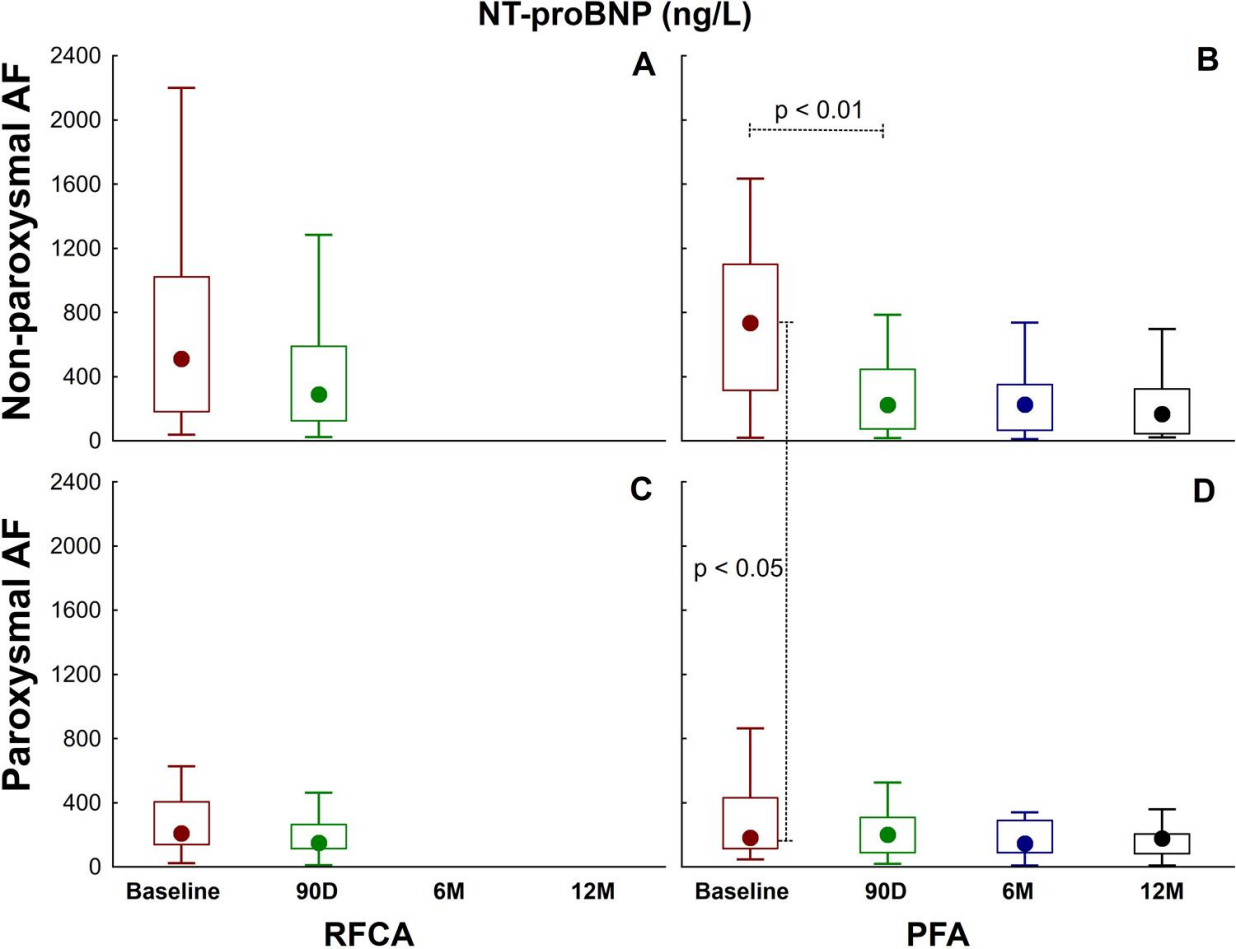
Dynamics of NT-proBNP, comparison of RFCA and PFA. AF, atrial fibrillation; NT-proBNP, N-terminal B-type natriuretic propeptide; NS, not significant; RFCA, radiofrequency catheter ablation; PFA, pulsed field ablation. The box-and-whisker plot shows the median, IQR, and range, excluding outliers.

The baseline NT-proBNP level was 313 (118; 864) ng/L in the PFA group. Compared with baseline, we observed a significant decrease in its level after 90 days (214 (81; 378), *p* = 0.04). The dynamics of NT-proBNP levels are shown in Figure 1D. When comparing patients by AF type, we observed higher baseline NT-proBNP levels in patients with non-paroxysmal AF [735 (314; 1,101) vs. 182 (114; 432) ng/L; *p* = 0.03]. Patients with non-paroxysmal AF showed a significant decrease in NT-proBNP (*p* = 0.006) after 3 months [90-day level was 224 (66; 351) ng/L]. In patients with paroxysmal AF, the difference was not significant (Figures 4B,D).

### 3.4 Comparison of GDF-15, hs-cTnT, and NT-proBNP between PFA and RFCA

PFA patients were compared with a historical cohort of 50 patients with a median age of 65 (57; 69) years, 64% of whom were males, who underwent RFCA. The baseline demographic and clinical data comparing the two groups are presented in Table 2. Compared with the RFCA cohort, PFA patients had significantly smaller LA volumes, lower HAS-BLED and ABC-bleeding scores, and fewer patients were medicated with amiodarone.

The primary monitored procedure data, along with their comparison to RFCA patients, are visualized in Table 3. When comparing different ablation methods, we noted a significantly longer median procedural time in the RFCA than in the PFA group [155 (130; 190) vs. 73 (60; 90) min; *p* < 0.001].

No significant difference in GDF-15 level was observed between all patients treated with RFCA and those treated with PFA at any time point. However, a within-subjects test revealed significantly different GDF-15 dynamics between the PFA and RFCA groups (Sphericity assumed F 4.82, *p* = 0.01; Greenhouse-Geisser test: F = 6.16, *p* = 0.007). A larger area under the GDF-15 curve over the first 24 h was found in PFA than in RFCA patients [4,205 (IQR 2,376; 8,772) vs. 193 (IQR -1,505; 2,941) ng·h/L], *p* < 0.001. The multivariate regression analysis of the determinants of the 24-hour increase in GDF-15 showed that only the PFA procedure was independently associated with GDF-15 change. Detailed results are in Table 4. When the area under the GDF-15 curve over the first 24 h was used, the PFA was also an independent variable. More details are in the Supplementary material (Supplementary Table S1). GDF-15 values in the late period (90 days and 6 months) did not differentiate between groups.

**Table 4:** Multivariate regression analysis of determinants of GDF-15 increase in 24 h.

Variable	Regression coefficient	95% CI	P
PFA	-263	-463 to 64	**0**.**01**
Body mass index (kg/m^2^)	8.3	-9.8 to 26.4	0.49
HAS-BLED score	65	-120 to 251	0.35
CHA_2_DS_2_-VASc score	63	-71 to 197	0.39
Indexed left atrial volume (mL/m^2^)	0.58	-13 to 14	0.93
Male (yes=0)	-14	-222 to 193	0.89
Non-paroxysmal AF (yes=1)	76	-105 to 257	0.41
Amiodarone (yes=1)	-58	-242 to 127	0.53

PFA, pulsed field ablation; AF, atrial fibrillation.

Bold values indicate statistically significant results (*p* < 0.05).

Significantly higher second (at the end of procedure) and third (24 h after ablation) GDF-15 levels were observed after PFA than RFCA in patients with paroxysmal AF: 963 (672; 1,266) vs. 630 (506; 804) ng/L with *p* < 0.001 and 1,362 (901; 1,960) vs. 843 (709; 1,195) ng/L, *p* = 0.003, respectively. More details are shown in Figures 2C,D. This difference was not recognized in non-paroxysmal subgroups (Figures 2A,B**)**. No correlation was found between GDF-15 levels and procedural data, including procedure duration, the extent of ablation, ablation time, or number of PFA applications.

Maximal documented values of hs-cTnT (24 h post ablation) were significantly higher in the PFA group than in the RFCA group in both paroxysmal and non-paroxysmal AF: 1,272 (992; 1,472) vs. 410 (265; 582) with *p* < 0.001 and 1,349 (1,129; 1,768) ng/L vs. 466 (341; 583) ng/L, *p* < 0.001, respectively. The comparison is visualized in Figure 3. A larger area under the hs-cTnT curve over the first 24 h was found in PFA than in RFCA patients [16,724 (13,885; 20,238) vs. 7,454 (4,224; 8,907) ng·h/L], *p* < 0.001.

No significant difference in NT-proBNP levels was found between the PFA and RFCA groups; the comparison is visualized in Figure 4.

## 4 Discussion

The main finding of our study is that PFA of AF is associated with significant changes in circulating GDF-15 levels. To our knowledge, this is the first study to describe the early and late dynamics of GDF-15 after PFA. Although peak levels were comparable between PFA patients and the historical RFCA cohort, the dynamics of GDF-15 in the early post-procedural period differed. Finally, PFA is associated with a significant elevation in hs-cTnT concentrations, without a corresponding change in GDF-15.

GDF-15 is a non-specific biomarker that is elevated in response to various forms of physiological stress, including inflammation, hypoxia, and tissue damage (12, 13). Its serum levels are commonly increased in individuals with cardiovascular conditions such as acute and chronic coronary syndromes, heart failure, and AF (14, 15). Several studies have demonstrated that GDF-15 levels increase in response to both surgical (16, 17) and catheter (18) procedures; however, this data is limited. Furthermore, elevated serum levels may help identify high-risk patients preoperatively before cardiovascular surgery (16). Additionally, the dynamics of changes in GDF-15 can also predict complications of procedures after cardiac surgery, such as heart failure and significant renal impairment (16). A recent meta-analysis further confirmed that elevated preprocedural GDF-15 levels predict adverse outcomes across various cardiovascular interventions (19). However, data on changes in GDF-15 levels in patients undergoing left atrial catheter ablation remain limited. We have demonstrated in a previous study that RFCA significantly affects GDF-15 levels, with distinct patterns of GDF-15 dynamics in paroxysmal and non-paroxysmal AF (11).

This study focused on changes in GDF-15 levels in patients treated with PFA. PF energy is a novel technology in catheter ablation of AF dedicated to pulmonary vein isolation. In contrast to thermal ablation, which causes coagulation necrosis that subsequently heals with a scar (18), PF uses high-energy, ultrashort electrical pulses to increase cell membrane permeability, leading to cell apoptosis. The ablation parameters are set to selectively ablate myocardial tissue (20, 21). In contrast to RFCA, PFA energy induces nonthermal cell death. PFA for AF has been shown to be a fast, safe, and effective method for pulmonary vein isolation with excellent durability (17). However, PFA is associated with elevations in markers of myocardial damage, platelet and coagulation activation, and erythrocyte destruction (10, 22). In this context, GDF-15 elevation post PFA was highly appreciated. The acute dynamics of GDF-15 levels correspond to acute injury caused by ablation, leading to the destruction of tissue responsible for arrhythmia, and subsequently reducing the incidence of AF (20, 21). The extension of ablation could be confirmed with a highly expected elevation of hs-cTnT as a marker of myocardial damage (23). Although no significant correlation was observed in the overall cohort, a weak positive correlation between GDF-15 and hs-cTnT levels was identified in patients with paroxysmal AF.

We speculate that this may be related to different biological mechanisms underlying the release of these biomarkers. Elevation of hs-cTnT after catheter ablation likely reflects myocardial injury induced by the ablation energy used, and may be related to the extent of the lesion (24). In our study, we observed significantly higher peak hs-cTnT levels in the PFA group than in the RFCA group. Evidence suggests that PFA creates larger or more complete lesions, particularly when using flower-shaped basket catheters that enhance contact and lesion uniformity (25). In contrast, GDF-15 is considered a nonspecific stress-responsive biomarker that may increase in response to inflammatory activation, oxidative stress, or tissue injury (13). However, a previous study did not find an association between other inflammatory markers (e.g., white blood count and high-sensitivity C-reactive protein) in patients with AF (26). The nonspecific elevation of GDF-15 is also supported by the lack of correlation with procedural characteristics (including procedural time, ablation extension, radiofrequency time, or number of PFA applications) and by differences in its levels between the ablation technologies used.

We have noticed differences in the area under the GDF-15 curve during the early post-ablation period between patients treated with PFA and RFCA. This observation seems to be in contradiction to the absence of differences in measured values at defined samplings. We suggest that the AUC method is more sensitive to slight changes in the sequence of values. The difference could also correspond to a decrease in GDF-15 values in the first post-ablation sample in RFCA (11). We only hypothesize that the greater relative increase in GDF-15 concentration in patients post PFA is more likely due to a stronger acute systemic stress response associated with the electroporation-based mechanism of myocardial injury. On the contrary, several clinically relevant baseline differences were also observed between the PFA and RFCA cohorts, including indexed left atrial volume, HAS-BLED score and amiodarone use. This finding is mitigated by multivariate regression analysis, which has shown that the type of ablation was an isolated independent variable that influenced the early dynamics of GDF-15. Due to retrospective design and a limited number of patients, the differences between PFA and RFCA must be interpreted as associations rather than evidence of the direct effect of ablation technology.

The greater relative increase in GDF-15 concentration in patients with paroxysmal AF treated with PFA, compared to RFCA, was found. In contrast, no such difference was observed in patients with persistent AF, which we hypothesize may be due to greater baseline variability and nonspecific elevation of GDF-15 associated with more advanced atrial remodeling and chronic inflammatory burden in this population, potentially masking acute procedure-related changes. This observation is consistent with previous findings showing that GDF-15 levels correlate with atrial structural remodeling, AF persistence, and arrhythmia recurrence following RFCA (26). Experimental and clinical studies suggest that GDF-15 is involved in fibrotic processes by promoting fibroblast proliferation and is positively associated with the extent of myocardial fibrosis (27, 28). These findings may suggest differences in the acute biomarker response between AF subtypes; however, given the limited sample size of the subgroup analyses, they should be interpreted with caution and require confirmation in larger or randomized cohorts.

In clinical practice, NT-proBNP is traditionally used as a marker of heart failure. However, its level is significantly influenced by the presence of AF and other arrhythmias and is therefore also considered a marker of good arrhythmia control (29). In this regard, it differs markedly from GDF-15, which appears to be more stable regardless of arrhythmia presence and may thus be interpreted independently of the current rhythm status. In our study, as expected, we observed a significant decrease in NT-proBNP levels three months after ablation. Furthermore, we recorded significantly higher values in patients with non-paroxysmal AF, which can be explained by the greater AF burden. However, when comparing different ablation modalities, no significant differences in NT-proBNP levels were observed, consistent with the findings above (29).

### 4.1 Clinical relevance

Biomarker GDF-15 is a routinely analyzed biomarker in many indications. Understanding its properties in response to various clinical situations is essential in daily clinical practice. In combination with other parameters, it has been introduced as a potent tool in detecting bleeding risk in anticoagulated patients (2) and the risk of death in patients with AF (3): the ABC bleeding and the ABC death score, respectively. The ABC-bleeding score is calculated based on five parameters: age, GDF-15, hs-cTnT, hemoglobin level, and bleeding history (2). In correlation with changes in GDF-15 and hs-cTnT levels, we observed a significant increase in the ABC bleeding score 24 h after the procedure for both ablation methods. The elevation of this score in the PFA group is explained by the higher increase in hs-cTnT levels. However, the ABC-bleeding score was originally developed for long-term bleeding risk prediction rather than for assessing immediate peri-procedural bleeding risk. Therefore, the transient increase observed shortly after ablation should be interpreted with caution. Importantly, no bleeding events were observed in our study population, which further limits the clinical interpretation of these short-term changes. The timing of biomarker sampling may influence the calculated ABC-bleeding score, as procedure-related increase in GDF-15 and hs-cTnT can transiently increase the score without necessarily reflecting a true change in bleeding risk. Therefore, its clinical relevance in the immediate post-ablation period is limited.

### 4.2 Limitations

This study has several limitations. First, this was a single-center, observational, non-randomized study with a relatively small number of patients, which may have limited the ability to detect small differences between groups, particularly in subgroup analyses. For some analyses, the study is underpowered with risk of type II error. Second, we cannot identify the exact timing of peak serum GDF-15 levels. As observed in the RFCA group, the maximal level may occur between 24 and 48 h after ablation, or even beyond 48 h. Third, the comparison between ablation technologies relied on a prospective PFA cohort and a historical RFCA cohort. Although both cohorts originated from the same center, the non-contemporaneous design may introduce potential sources of bias related to differences in patient selection, procedural strategies, or peri-procedural management over time. Even though multivariate regression analysis has shown that the type of ablation was an isolated independent variable that influenced the early dynamics of GDF-15, several potentially clinically relevant baseline differences were observed between the PFA and RFCA cohorts. Finally, due to the limited number of clinical events, the present study was not powered to assess the relationship between GDF-15 dynamics and clinically relevant outcomes, such as bleeding events, AF recurrence, hospitalization, or mortality.

### 4.3 Generalizability

Despite these limitations, our findings provide novel information on the dynamics of GDF-15 after PFA of AF. Although the study was conducted at a single center, the patient population and procedural approach are comparable to those used in contemporary electrophysiology practice, which supports the potential generalizability of our findings to similar clinical settings.

## 5 Conclusions

GDF-15 levels changed significantly after PFA of AF. Its values and dynamics seem to be dependent on the type of ablation and the persistence of AF. PFA is associated with advanced elevation of hs-cTnT. The study is hypothesis-generating.

## Data Availability

The original contributions presented in the study are included in the article/Supplementary Material, further inquiries can be directed to the corresponding author.
