# Supplementary material for: Growth differentiation factor 15 level changes in the early and late periods after radiofrequency and pulsed field catheter ablation of atrial fibrillation
Source: Front Cardiovasc Med. 2026 Aug 5;13:1798578. doi: 10.3389/fcvm.2026.1798578 (PMC13485757; doi:10.3389/fcvm.2026.1798578)
Supplement: Supplementary file 1 [file Table1.docx]

**Table S1: Multivariate regression analysis of determinants of Area Under the GDF-15 Curve over the first 24 hours.**

Note: PFA – pulsed field ablation; AF – Atrial fibrillation.

| **Variable** | **Regression coefficent** | **95% CI** | **P** |
| --- | --- | --- | --- |
| **PFA** | -4392 | -7177 to -1606 | **0.002** |
| **Body mass index (kg/m^2^)** | 139 | -114 to 391 | 0.49 |
| **HAS-BLED score** | 716 | -192 to 194 | 0.35 |
| **CHA_2_DS_2_-VASc score** | -96 | -1973 to 1780 | 0.39 |
| **Indexed left atrial volume (ml/m^2^)** | 1.34 | -192 to 194 | 0.93 |
| **Male (yes=0)** | -268 | -1363 to 2626 | 0.89 |
| **Non-paroxysmal AF (yes=1)** | 1311 | -1219 to 3840 | 0.41 |
| **Amiodarone (yes=1)** | -684 | -3261 to 1892 | 0.53 |
